# Supplementary material for: Effects of Physical Exercise on the Physical and Mental Health of Family Caregivers: A Systematic Review
Source: Healthcare (Basel). 2025 May 20;13(10):1196. doi: 10.3390/healthcare13101196 (PMC12111804; doi:10.3390/healthcare13101196)
Supplement: Supplementary file 1 [file healthcare-13-01196-s001.zip › healthcare-3613494-supplementary.pdf]

SUPPLEMENTAL MATERIAL. Effects of Therapeutic Physical Exercise on the Physical and Mental Health of Family Caregivers: A Systematic Review

S1. Summary of the study characteristics and main results.

**Author(s), year, country**	Participants characteristics (number, gender, mean age, relationship to dependent)	Intervention (type, duration, frequency, intensity) and comparison (control group)	Measures	Outcomes
Madruga et al., 2021 Spain [25]	N=48<br>Female<br>61 years old<br>wives	Intervention: Physical exercise program home based<br>Duration: 36 weeks<br>Frequency: 2 sessions/ week, 60 min/ session<br>Intensity: moderated<br>Comparation: habitual activities	The subjective burden: Zarit Burden Interview<br>The risk of depression: Geriatric Depression Scale Short Form 15-item version<br>Psychological symptoms: Symptom Check List-90-Revised (SCL-90-R)	Positive impact on caregivers' subjective burden and risk of depression
Connell CM et Janevic NR, 2009 USA [26]	N=137<br>female<br>67 years old<br>wives.	Intervention: telephone-based exercise<br>Duration 6 months<br>Frequency: 30 min/session 3 session/week<br>Intensity: low to moderate<br>Comparation: not intervention	Perceived stress: Cohen Perceived Stress Scale<br>Self-efficacy: Self-Rated Abilities for Health Practices	Reductions in perceived stress, increases in exercise self-efficacy

Kim et al., 2023<br>South Korea<br>[27]	N=64<br>Female<br>60 years old<br>relatives	Intervention: physical activity program<br>Duration: 8 weeks<br>Frequency: 60 minutes/session, 1 session/week<br>Intensity: not available<br>Comparation: not intervention	Self-efficacy: Self-Rated Abilities for Health Practices<br>Physical function Short Physical Performance Battery<br>Caregiving burden: Zarit Burden Interview<br>Depressive symptom: Geriatric Depression Scale Short Form	Improvements in self-efficacy, physical function and quality of life related to health; decrease in caregiving burden and depressive symptoms
Lök et al., 2023<br>Turkey<br>[28]	N=60<br>female<br>50 years old<br>relatives	Intervention: Physical Activity Program<br>Duration: 8 weeks<br>Frequency: 1 session/ week, 60 min/session<br>Intensity: moderated<br>Comparation: normal routine	Burden: Zarit Caregiver Burden Scale<br>Healthy lifestyle behavior: Healthy Lifestyle Behavior Scale	Improvements in lifestyle and burden
Hives et al., 2021<br>Canada<br>[29]	N=68<br>most female<br>61 years old<br>daughters and wives mostly	Intervention: Aerobic exercise program<br>Duration: 24 weeks<br>Frequency: 3 sessions/ week, 20 min/session,<br>Intensity: in increase at week 9<br>Comparation: normal routine	Burden: Zarit Caregiver Burden Scale<br>Depressive symptomatology: Patient Health Questionnaire	Decrease in burden and in reported depressive symptomatology
Lin et al., 2023<br>USA<br>[30]	N=76<br>92% female<br>56 years old<br>most daughters	Intervention: social app to promote physical activity and well-being with daily step<br>Duration: 6 weeks<br>Frequency: daily<br>Intensity: light and moderate to vigorous<br>Comparation: Another version of the app without social contact	Stress: The Caregivers' Stress Scales<br>Physical activity: Fitbits<br>Social support: Lubben Social Network Scale	Improvements in well-being and social support

Puterman et al., 2018 Canada [31]	N=68<br>most female<br>63 years old<br>50% daughters, 46% spouses, 4% relatives	Intervention: physical activity program<br>Duration: 24 weeks<br>Frequency: 40 min/session, 3–5 sessions/week. Intensity: Moderate to vigorous<br>Comparation: Waitlist	Perceived stress: Cohen Perceived Stress Scale	Reduction in perceived stress and an increase in cardiorespiratory fitness
Castro et al., 2002 USA [32]	N=100<br>female<br>62 years old<br>relatives	Intervention: Physical activity home-based<br>Duration: 12 months<br>Frequency: 30 min/session, 4 sessions/week<br>Intensity: moderated<br>Comparation: Nutrition education	Stress: Cohen Perceived Stress Scale<br>Perceived social support: Interpersonal Social Evaluation List Anxiety: Taylor Manifest Anxiety Scale<br>Burden: Screen for Caregiver Burden	Improvement in perceived stress, burden, and depression
Yilmaz et al., 2019 Turkey [33]	N=44<br>most female<br>50 years old<br>relatives	Intervention: progressive muscle relaxation exercises at home<br>Duration: 8 weeks<br>Frequency: 28 min/day, 3 days/week<br>Intensity: not available<br>Comparation: nonintervention	Caregiver burden: Zarit Caregiver Burden Scale<br>Depression: Beck Depression Scale	Decrease in the caregiver burden and level of depression
Montero-Cuadrado et al., 2020 Spain [34]	N=68<br>Female<br>64 years old<br>48% daughters, 37% spouses, 15% relatives	Intervention: Family caregiver care program (FCCP) and physical therapeutic exercise (PTE)<br>Duration: 12 weeks<br>Frequency: FCCP (4 sessions, 6 h/session) + PTE (60 min/session, 3 sessions/week, 36 sessions)<br>Intensity: not available<br>Comparation: FCCP (4sessions, 6h).	SF-36 Spanish version scale	Improvement of quality of life, subjective burden, anxiety, depression and health-related physical condition

Prieto-Prieto et al., 2022<br>Spain<br>[35]	N= 48<br>female<br>60 years old<br>most daughters	Intervention: Physical exercise intervention home-based (aerobic exercises)<br>Duration: 9 months<br>Frequency: 1h/session, 2 sessions/week<br>Intensity: moderate<br>Comparation: Normal daily activities.	Caregiver´s subjective burden: Zarit Burden Interview<br>Physical fitness: battery of health-related fitness tests for older people	Improvement in the dimensions of general health, vitality and mental health
King et al., 2002<br>USA<br>[36]	N= 100<br>Female<br>65 years old<br>relatives	Intervention: exercise training home-based telephone-supervised<br>Duration: 12 months<br>Frequency: 30-40 min/session, 4 sessions/week<br>Intensity: moderate<br>Comparation: Nutrition education	The sleep quality: Pittsburgh Sleep Quality Index<br>Psychological distress: Perceived Stress Scale	Improvement in sleep quality and psychological distress
Flanagan et al., 2022<br>USA<br>[37]	N= 32<br>most female<br>57 years<br>relatives	Intervention: Walking intervention<br>Duration: 8 weeks<br>Frequency: 30 min/day, 5 days/week<br>Intensity: not available<br>Comparation: Normal daily activity	Well-being: Short Warwick–Edinburgh Mental Well-being Scale<br>Perceived stress: Perceives Stress Scale	Improvement in walked well-being and perceived stress
Hirano et al., 2011<br>Japan<br>[38]	N= 31<br>most female<br>74 years old<br>relatives	Intervention: Regular exercise<br>Duration: 12 weeks<br>Frequency: 3 sessions/week<br>Intensity: moderate<br>Comparation: Non exercise	Burden: Zarit Caregivers Burden Scale<br>Physical activity: Physical activity Questionnaire Score for the elderly	Reduction in the burden and in the feeling of fatigue; improvement in quality of sleep
Farran et al., 2016<br>USA<br>[39]	N= 211<br>most female<br>61 years old<br>daughters or wives	Intervention: physical activity at home<br>Duration: 12 months<br>Frequency: ≥ 150 min/weekly<br>Intensity: moderate to vigorous<br>Comparison: Caregiver Skill Building	Physical function: 2 min Step Test and the 30s Chair Stand Test.	Increase in physical activity and the number of steps maintained stable caregiving hours

Gary et al., 2020<br>USA<br>[40]	N=127<br>Most female<br>55 years old<br>Spouse partner or other adult Family member	Intervention: Aerobic and resistance exercise program home-based and psychoeducation program<br>Duration: 6 months<br>Frequency: 4 sessions/week, 12 weeks and 12 weeks maintenance period<br>Intensity: progressive low to moderate<br>Comparation: psychoeducation alone and usual care attention control group	Physical function: 6-min walk test, handgrip, and upper and lower strength<br>Caregiving perceptions: Bakas Caregiving Outcomes Scale	Improvements in physical function and caregiver perception
Loi et al., 2024<br>Australia<br>[41]	N=121<br>82% female<br>70 years old<br>78% spouse	Intervention: Physical activity home based<br>Duration: 6 months<br>Frequency: 30 min/day, 5 days/week<br>Intensity: individualized for each patient<br>Comparation: Semi-structured discussions about any topics of interest	Depressive symptoms: GDS-15 and Zarit burden interviews<br>Independence: Modified Barthel Index<br>Cognition: Standardized Mini-Mental State Examination	Reductions depressive symptoms

S2. Search strategy

Embase:

('physiotherapy'/exp OR physiotherapy OR 'physical activity'/exp OR 'physical activity') AND ('caregiver'/exp OR caregiver)

PubMed:

(("Exercise"[Mesh]) OR "Physical Therapy Modalities"[Mesh]) AND "Caregivers"[Mesh]

CINALH:

(MH "Exercise+") OR (MH "Physical Therapy+") OR (MH "Physical Activity") AND (MH "Caregivers")

Scopus

(Exercise OR "Physical Therapy Modalities" AND Caregiver
